# Characterizing operant hyperactivity in the Spontaneously Hypertensive Rat

**DOI:** 10.1186/1744-9081-8-5

**Published:** 2012-01-26

**Authors:** Jade C Hill, Katrina Herbst, Federico Sanabria

**Affiliations:** 1 Department of Psychology, Arizona State University, P.O. Box 871104, Tempe, Arizona 85287-1104, USA

**Keywords:** ADHD, Spontaneously Hypertensive rat, hyperactivity, operant, variable interval, bout

## Abstract

**Background:**

Operant hyperactivity, the emission of reinforced responses at an inordinately high rate, has been reported in children with ADHD and in the Spontaneously Hypertensive Rat (SHR), the most widely studied animal model of ADHD. The SHR emits behavior at hyperactive levels, relative to a normoactive strain, only when such behavior is seldom reinforced. Because of its dependence on rate of reinforcement, operant hyperactivity appears to be driven primarily by incentive motivation, not motoric capacity. This claim was evaluated in the present study using a novel strategy, based on the organization of behavior in bouts of reinforced responses separated by pauses.

**Method:**

Male SHR, Wistar-Kyoto (WKY) and Wistar rats (WIS) were exposed each to a multiple variable-interval schedule of sucrose reinforcement (12, 24, 48, 96, and 192 s) between post-natal days (PND) 48 and 93. Responding in each schedule was examined in two epochs, PND 58-62 and 89-93. Parameters of response-reinforcement functions (Herrnstein's hyperbola) and bout-organized behavior were estimated in each epoch.

**Results:**

SHR emitted higher response rates than WKY and WIS, but only when rate of reinforcement was low (fewer than 2 reinforcers per minute), and particularly in the second epoch. Estimates of Herrnstein's hyperbola parameters suggested the primacy of motivational over motoric factors driving the response-rate differential. Across epochs and schedules, a more detailed analysis of response bouts by SHR revealed that these were shorter than those by WKY, but more frequent than those by WKY and WIS. Differences in bout length subsided between epochs, but differences in bout-initiation rate were exacerbated. These results were interpreted in light of robust evidence linking changes in bout-organization parameters and experimental manipulations of motivation and response-reinforcement contingency.

**Conclusions:**

Operant hyperactivity in SHR was confirmed. Although incentive motivation appears to play an important role in operant hyperactivity and motoric capacity cannot be ruled out as a factor, response-bout patterns suggest that operant hyperactivity is primarily driven by steeper delay-of-reinforcement gradients. Convergence of this conclusion with theoretical accounts of ADHD and with free-operant performance in children with ADHD supports the use of SHR as an animal model of ADHD.

## Background

Attention deficit hyperactivity disorder (ADHD) is the most commonly diagnosed childhood psychiatric disorder, affecting between 2% and 12% of grade school children, and around 4% of adults [1-4]. It is characterized by difficulties related to impulsivity, inattention, and hyperactivity [5]. ADHD is associated with problems in school, poor interpersonal relationships, and psychological problems such as depression and anxiety, among others [6-9].

The Spontaneously Hypertensive Rat (SHR) is the most widely used animal model of ADHD [10-12]. Evidence suggests that SHR displays the three main behavioral characteristics of ADHD: impulsivity [13-15], inattention [16], and hyperactivity [17]. Nonetheless, the reliability of some of this evidence and the validational support it provides has been disputed [18,19]. Sanabria and Killeen [13] addressed the inconsistency of the evidence regarding response inhibition deficits in SHR. They concluded that, without a model of response inhibition and appropriate procedures for estimating model parameters, claims about impulsivity in SHR are unlikely to converge. The purpose of this paper is to extend this reasoning to another symptom of ADHD that is presumably expressed in the SHR: hyperactivity.

Hyperactivity in SHR has been assessed using the open-field method and the operant-conditioning method. The open-field method consists of measuring the amount of locomotor activity (typically in the form of infrared beam breaks) in an enclosure [20]. Because activity measured by this method does not yield programmed consequences, we refer to it as *spontaneous *activity. The operant-conditioning method consists of measuring the rate of emission of a target response (typically lever pressing), where the target response occasionally produces a reinforcer (typically food). Because activity measured by this method operates on a specific feature of the environment and yields a programmed consequence, we refer to it as *operant *activity. Operant activity is often observed under interval schedules of reinforcement, in which only the first target response following a programmed interval is reinforced. Interval schedules maintain an approximately constant rate of reinforcement regardless of response rate [21], thus isolating changes in rate of reinforcement from changes in activity.

It is not clear that the SHR displays more spontaneous activity than control strains. Whereas some research has demonstrated spontaneous hyperactivity in the SHR [22-27], other research has shown this effect only at certain ages [19,28], and still other research has not shown such an effect [29-31]. In contrast, operant hyperactivity is well demonstrated in the SHR [16,25,31-34]. Under interval schedules of reinforcement, the SHR typically responds at significantly higher rates than control strains [16,17]. An analogous difference has been observed between children with and without ADHD [35].

Performance under varying rates of reinforcement has been informative of the nature of operant hyperactivity in the SHR. Response rates in the SHR and control strains covary with rate of reinforcement, but the SHR responds at abnormally higher rates only when rate of reinforcement is low [32,33]. These researchers showed that maximal responding was about equal for SHR and Wistar-Kyoto rats (WKY, which typically serves as control strain), suggesting that superior motor ability alone cannot explain hyperactivity in the SHR. High rates of reinforcement have also been shown to normalize the operant performance of children with ADHD [36,37]. These results suggest that non-motoric processes, such as differences in responsiveness to incentives [38], may contribute to operant hyperactivity. The purpose of the present study is to advance the identification of such processes by examining SHR performance at a high level of detail.

**Figure 1:**
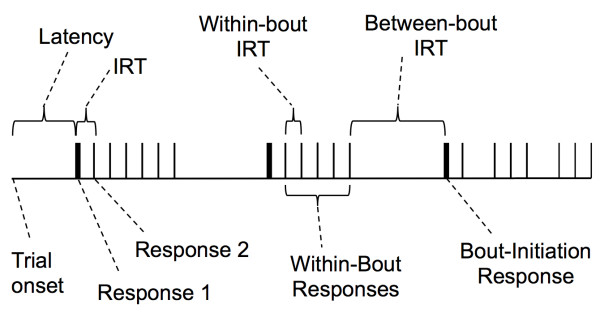
**Schematic timeline depicting the bout-and-pause model of free-operant performance**. A trial starts at the left-end of the timeline and progresses to the right. The time between trial onset and the first response (vertical line labeled "Response 1") is the latency for that trial. The time between two consecutive responses is an inter-response time, or IRT. There are two types of IRTs: within-bout (short) and between-bout (long). Thicker vertical lines are bout-initiation responses; thinner lines are within-bout responses. The length of a bout is the number of responses between latency and the first between-bout IRT, or between two between-bout IRTs.

Inferences on motor and motivational processes have been drawn from performance in variable interval (VI) schedules of reinforcement. In these schedules, the first response following an unsignaled interval of variable duration is reinforced. Inferences are based on parameters of models fit to average response rates in VI schedules [39,40]. Not all responses in VI schedules, however, are functionally equivalent, so averaging all responses in a session may neglect useful information [41]. In fact, the distribution of inter-response times (IRTs) provides additional information about the multiple sources of variance in VI performance [42-47]. Figure 1 illustrates the IRT model used in the present study, which we call the bout-and-pause model. Each vertical line represents a response; the spaces between vertical lines represent the IRTs. The critical assumption of this model is that operant responding occurs in bouts separated by relatively long pauses [40,41,48]. Thus, increased responding in the SHR at low rates of reinforcement may be due to (1) faster responding within bouts, (2) longer bouts, or (3) shorter pauses between bouts. The purpose of this study was to replicate past results that show that SHR hyperactivity is constrained to low rates of reinforcement, and to characterize operant hyperactivity in SHR in terms of bout-and-pause parameters.

## Methods

### Subjects

Eighteen male rats, 6 each of three different strains were obtained from Charles River Laboratories (US) (Spontaneously Hypertensive Rats, SHR; Wistar rats, WIS) and Harlan Laboratories (US) (Wistar-Kyoto rats, WKY). The substrain of WKY bred by Harlan Laboratories has been shown to be the most appropriate normoactive control strain for the SHR [49].

Rats arrived to the laboratory on post-natal day (PND) 24-25. Hopper training commenced on PND 39. Rats were pair-housed within their strains in a colony room with a 12:12 hour light:dark cycle; experiments were conducted during the dark cycle. Rats were maintained at 85% of their free-feeding weights based on a logistic function fitted to the growth curves provided by breeders. All rats were handled for a minimum of 2 min/day by the researchers in the days preceding hopper training. Animals were weighed every morning, and fed a supplementary amount of rodent chow every evening, at least 12 hr before the following experimental session. At the beginning of operant training the mean weights for the SHR, WKY, and WIS were 118, 127, and 190 g, respectively. Home cages were always equipped with water bottles. All handling procedures in the present study were maintained according to the guidelines of the National Institute for Health, which were approved by the Arizona State University Institutional Animal Care and Use Committee.

### Apparatus

All experimental sessions were conducted in 6 MED Associates^® ^modular test chambers (305 mm × 241 mm × 210 mm). Each sound- and light-attenuating box contained a ventilating fan. The fan provided a masking noise of about 60 dB. The bottom of each box was lined with a catch pan full of sanitary chip litter, and the floor of each chamber had thin metal bars. The front and back walls and the ceiling were made of clear polycarbonate; the front wall also served as a door. The food receptacle was attached to a square aluminum aperture (51 mm sides, 15 mm above the chamber floor), centered on the side wall against which the chamber door was latched. Activation of the food dispenser released one 45-mg food pellet (Dustless Precision Pellets^®^, Rodent Grain-Based Diet, Bio-Serv, Frenchtown, NJ). Although the study only involved use of the right lever (closest to the door of the chamber), two retractable levers (MED associates, ENV-112CM) were on either side of the food hopper. The inside edge of each lever was 8 mm from the closest vertical edge of the receptacle. A Med-PC^® ^interface connected to a PC computer ran Med-PC IV^® ^software. This computer recorded lever presses when a force of about 0.2N was applied to the lever. Responses were recorded with a 110-ms resolution. The house light was not turned on for experimental sessions. A speaker located at the top of the side wall opposite to the food receptacle emitted 75 dB tones from a generator (ENV-223).

### Procedure

#### Hopper training and autoshaping

Experimental sessions were conducted daily at approximately the same time of day for each rat. Hopper training consisted of presenting a food pellet every 15 s on average. After two days, rats were eating consistently from the hopper and autoshaping of lever pressing started. Autoshaping consisted of extending a randomly selected lever (left or right) for 10 s, every 30 s; a food pellet followed only right lever retractions. After 9 days rats were consistently pressing the right lever each time it was extended.

#### Operant Task

The first five minutes of each session served as an acclimation period, during which the houselight was off and the levers were retracted. After the acclimation period, the right lever was extended into the chamber, and a multiple variable interval (VI) schedule was in effect. One of five VI schedules (VI 12, 24, 48, 96, or 192 s) was randomly selected. Schedules were implemented on each trial by selecting without replacement from an 8-item Fleschler-Hoffman distribution of intervals [50]; the mean of the distribution was the nominal VI requirement. Responses that occurred during the interval were recorded but had no programmed effect. Once the selected interval time elapsed, the first lever press resulted in the delivery of one food pellet into the feeding aperture, which served as reinforcement. After each pellet delivery, the lever was retracted, a 5-s inter-trial interval (ITI) ensued, then the lever was extended again and another interval was selected from the same VI distribution. When an 8-item distribution was exhausted, the ITI was 20 s and another VI schedule was selected. The five schedules of reinforcement were signaled by one of five tones. Each tone (3-12 kHz) was presented on a unique on:off cycle (200-1000 ms) for the duration of the schedule. Sessions ended when every schedule was implemented once, or after 70 minutes, whichever happened first. Fifty-four daily sessions were conducted, 7 days/week.

#### Measures

The first analysis was based on two measures: response rate and reinforcement rate. Response rate was computed for each VI schedule as the number of responses emitted while the schedule was effective, divided by the time the schedule was in effect (excluding ITIs). Reinforcement rate was computed for each VI as the number of reinforcers collected at each schedule, divided by the time the schedule was in effect (excluding ITIs). Response rates were measured in two epochs, PND 58-62 (epoch 1) and PND 89-93 (epoch 2), following approximately 10 and 40 sessions of VI training, respectively. Epoch 1 corresponds to a conservative estimate of early adulthood, but possibly captures late adolescence. Epoch 2 corresponds to adulthood [51]. Herrnstein's (1970) hyperbola parameters were estimated on the basis of response rates (explained in *Results *section) [40].

In a subsequent analysis, response rates were further analyzed by separating response latencies from inter-response times (IRTs). The distinction between latencies and IRTs is depicted in Figure 1. Latencies were the intervals between trial onset (lever extension) and the first lever press in that trial. IRTs were the intervals between consecutive lever presses within the same trial. Latencies were classified in two groups: the first latency in each VI (Latency 1), and all subsequent latencies within the same VI (Latencies 2-8). This classification took into account that, within each VI schedule, the duration of the first interval to reinforcement could only be cued by the discriminative tone, whereas the duration of subsequent intervals could also be cued by the duration of the preceding intervals. Median Latencies 1 and 2-8 were computed separately for each rat within each VI schedule and epoch, and then averaged within strain (mean median latencies). Estimates of bout-initiation rates, within-bout response rates, and bout length-the parameters of the bout-and-pause model-were based on the distribution of IRTs (explained in *Results *section).

## Results

**Figure 2:**
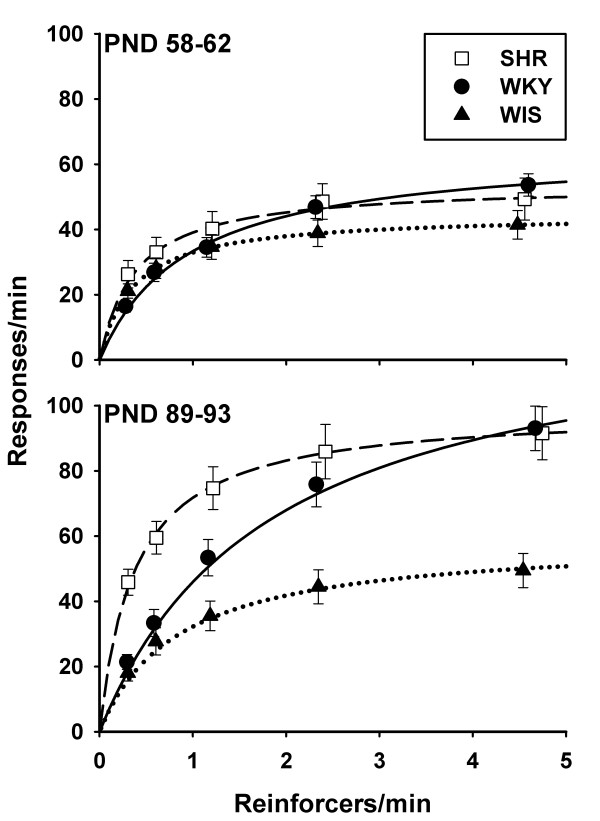
**Mean (± SEM) response rates of each strain (SHR: unfilled squares; WKY: filled circles; WIS: filled triangles) as a function of mean rate of reinforcement, in two epochs: PND 58-62 (epoch 1; top panel) and PND 89-93 (epoch 2; bottom panel)**. Response rate increased with rate of reinforcement in all strains and epochs. SHR response rates were higher than those of WKY and WIS when reinforcement was delivered less than twice per minute. At higher rates of reinforcement, WIS response rates were lower than those of SHR and WKY. Curves through the data are traces of Herrnstein's hyperbola (Equation 1).

Figure 2 shows mean (± SEM) response rates of each strain as a function of rate of reinforcement in each epoch. Response rates of SHR are indicated by unfilled squares, WKY by filled circles, and WIS by filled triangles. Visual inspection of Figure 2 reveals a positive correlation between response rate and rate of reinforcement in all strains. Differences in response rates between strains and across rates of reinforcement are visible in both epochs. When rates of reinforcement were low (fewer than 2 responses per minute), SHR responded at a higher rate than other strains. At higher rates of reinforcement, SHR and WKY response rates converged, and WIS response rates remained low (40-50 responses per minute). These patterns of response rate across strains and schedules were visible in epoch 1 and were magnified in epoch 2. SHR response rate increased with age regardless of rate of reinforcement, whereas for WKY age-dependent increases in response rate were more noticeable at higher rates of reinforcement, and for WIS there was virtually no change in response rate with age. To characterize these patterns of response rate, we estimated the parameters of Herrnstein's (1970) hyperbola and compared them across strains [40].

### Herrnstein's hyperbola

Herrnstein (1970) extended the Matching Law [52] to describe the relation between response rate (*B*) and rate of reinforcement (*R*) on a single operandum. Herrnstein's rationale was that all the responses other than the target response are reinforced at an unknown rate. Such rate, however, may be estimated if it is assumed that (a) the ratio of two response rates matches the ratio of the corresponding reinforcement rates (Matching Law), and (b) the target response rate and the non-target response rate add to a constant *k*. Under such assumptions,

(1) $$B=\frac{kR}{R+R_{e}},$$

where *R_e _*is the estimated rate of reinforcement provided by non-target responses. When reinforcement is programmed on VI schedules, *R *typically falls only slightly below programmed reinforcement rates and thus serves as the independent measure; *B *is the dependent measure; *k *and *R_e _*are free parameters. Equation 1 predicts that responding increases at a negative pace as reinforcement increases, with asymptote *k. R_e _*is the rate of reinforcement at which response rate reaches half of its asymptote (i.e., when *R *= *R_e_, B *= *k */2).

Following Herrnstein's (1970) rationale [40], *k *is often interpreted as a maximum limit on motoric performance, influenced only by response characteristics; *R_e _*is interpreted as indexing motivation for the reinforcer, influenced only by reinforcer characteristics [39]. A large body of evidence supports Equation 1 as an accurate characterization of response-reinforcement functions like those in Figure 2[53-56]. The empirical support for motoric/motivational interpretations is, however, somewhat mixed [57].

Parameters of Equation 1 were estimated by fitting Equation 1 to the data of each individual animal, in each epoch, using the method of least squares. Parameters *k *and *R_e _*were assumed constant across values of *R*, but could vary between rats, thus yielding 2 × 18 = 36 model parameters. Comparisons were conducted between *mean *estimates of each strain, henceforth referred to by the parameter and strain abbreviation: *k*_SHR_, *k*_WKY_, *k*_WIS_, *R*_*e*SHR_, *R*_*e*WKY_, and *R*_*e*WIS_. The curves in Figure 2 are traces of Equation 1 using the mean estimates of *k *and *Re *for each strain.

Various constraints were imposed on model parameters to draw inferences on between-strain differences. These constraints consisted of holding constant the mean estimate of either model parameter across all, some, or none of the strains. Each particular combination of constraints constituted a hypothesis. Thus, for example, *k*_SHR _≠ *k*_WKY _= *k*_WIS_, *R*_*e*SHR_= *R*_*e*WKY_= *R*_*e*WIS_is the hypothesis that mean *k *varied between SHR and WKY, but not between WKY and WIS, and mean *R_e _*did not vary between strains. There were 15 possible constraint combinations.

Hypothesis testing was conducted separately in each epoch, using the corrected Akaike Information Criteria (AICc) [58],

(2) $$\text{AICc}=n\text{ln}\;\left(\text{RSS}/n\right)+\frac{2nc}{n-c-1}$$

where *n *is the number of observations (*n *= 5 schedules × 18 rats = 90 observations in each epoch), RSS is the minimized residual sum of squares obtained from fitting a hypothesis to the data, and *c *is the number of free parameters in the hypothesis. *c *can also be computed as the degrees of freedom of the estimates of the overall means of *k *and *R_e _*plus 1 parameter for error variance, i.e., 2 parameters × 18 rats - the number of constraints + 1. In the preceding example, *c *= 36 - 3 + 1 = 34 free parameters.

Note that AICc increases with RSS and with *c*; therefore smaller AICc are indicative of close fit to the data and parsimony. Hypotheses with smaller AICc were favored over those with higher AICc. ΔAICc*_i _*was computed as the difference between each the AICc of hypothesis *i *and the lowest AICc among all hypotheses (ΔAICc*_i _*= AICc*_i _*- AICc_MIN_). The hypothesis with fewest free parameters among those with ΔAICc < 4 was selected as the best description of the data. This selection was conducted separately for the 2 epochs in which data were collected.

**Figure 3:**
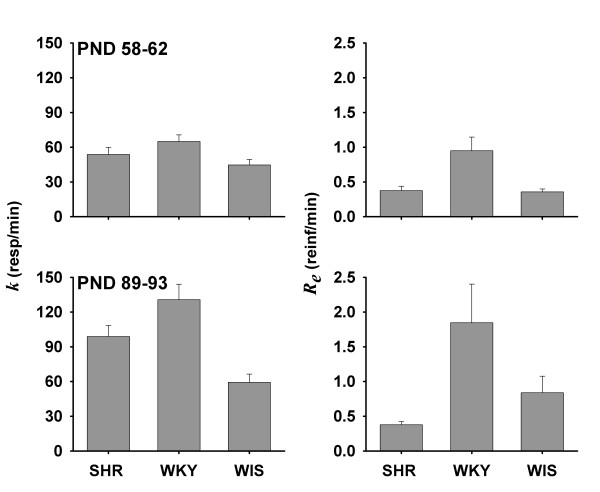
**Mean (± SEM) estimates of Herrnstein's hyperbola parameters *k *(asymptotic target response rate; left panels) and *R_e _*(rate of reinforcement of non-target behavior; right panels) for each strain in epochs 1 and 2 (top and bottom panels, respectively)**. Estimates are based on hypotheses selected according to AICc (Table 1). Estimates of *k *for SHR were intermediate relative to other strains. Estimates of *R_e _*for SHR were low relative to other strains, and approximately constant across epochs.

Table 1 shows the 5 hypotheses with the lowest ΔAICc in each epoch. The selected hypothesis for epoch 1 assumes that *R*_*e*WIS_= *R*_*e*SHR_, and all other parameters varied between strains. For epoch 2, the selected hypothesis assumes different parameters for each strain. Figure 3 shows the mean estimates of *k *and *R_e _*for each epoch based on the selected hypotheses. It was inferred that *k*_WKY _>*k*_SHR _>*k*_WIS _in both epochs, which indicates that WKY had the highest asymptotic response rates, followed by the SHR and then WIS. Mean *k *estimates increased across epochs for all strains. In epoch 1, *R*_*e*WKY_>*R*_*e*WIS_= *R*_*e*SHR_; at epoch 2, *R*_*e*WKY_>*R*_*e*WIS_>*R*_*e*SHR_. *R*_*e*SHR_does not appear to change across epochs, whereas *R*_*e*WKY_and *R*_*e*WIS_increased.

**Table 1:** Hypotheses of VI performance with lowest ΔAICc.

Hypothesis	*c*	RSS	ΔAICc
Epoch 1 (PND 58-62)			
*R*_*e*SHR_= *R*_*e*WIS_	36	306.78	0.00
*None*	37	306.12	5.49
*k*_WIS _*= k*_WKY_	36	458.24	36.11
*k*_SHR _= *k*_WIS_, *R*_*e*WIS _= *R*_*e*WKY_,	35	496.05	37.78
*k*_SHR _= *k*_WIS_	36	495.73	43.19
Epoch 2 (PND 89-93)			
*None*	37	581.35	0.00
*R*_*e*WIS _= *R*_*e*WKY_	36	738.84	15.89
*k*_SHR _= *k*_WIS_, *R*_*e*WIS _= R_*e*WKY_,	35	837.20	21.67
*k*_SHR _= *k*_WIS_	36	807.35	23.87
*R*_*e*SHR_= *R*_*e*WIS_	36	904.42	34.09

*Note*. The label of each hypothesis stipulates the constraints on mean parameter estimates. *c *is the number of free parameters in each hypothesis; RSS is the residual sum of squares from model fitting. For all hypotheses, the number of observations was *n *= 90. See Equation 2 and text for computation of ΔAICc. Hypotheses are arranged according to ΔAICc within each epoch. Hypotheses with ΔAIC = 0 were selected for parameter estimation.

Inferences from Herrnstein's hyperbola parameters suggest that instrumental overactivity could be attributed to a higher motivation for the reinforcer, which did not decline over nearly 30 days that separated the two assessment epochs, and not to differences in motoric capacity. This analysis, however, was based on average response rates in each VI schedule, which conflate two types of intervals within the denominator: response latencies and inter-response times (IRTs). Because rodent VI performance is typically organized in bouts [42], IRTs may be further disaggregated into between-bout and within-bout IRTs. Latencies, between- and within-bout IRTs may each depend on a distinct set of variables [45], which may further inform the sources of SHR overactivity.

In the next two sections we examine the components of response rate in SHR, WKY, and WIS. This analysis is aimed at identifying candidate components that may account for the differences in response rate between SHR and WKY selectively at low rates of reinforcement, and between SHR and WIS at all rates of reinforcement. An AIC-based analysis appears to be best suited to address this goal, because the relation between response rate and its components is not linear (see Appendix). Without an *a priori *selection of hypotheses, however, the combinatorial of parameters and factors implies a computationally intractable analysis that may ultimately select an unintelligible model [58]. Conventional approaches, such as null-hypothesis testing, are not designed for this task: falsifying the null hypothesis that a particular component did not vary between strains in one or more schedules provides little information on the contribution of that component to differences in response rate. Therefore, the analysis presented here is qualitative; inferences drawn from this analysis should be taken as exploratory and provisional, pending empirical verification.

### Latencies

**Figure 4:**
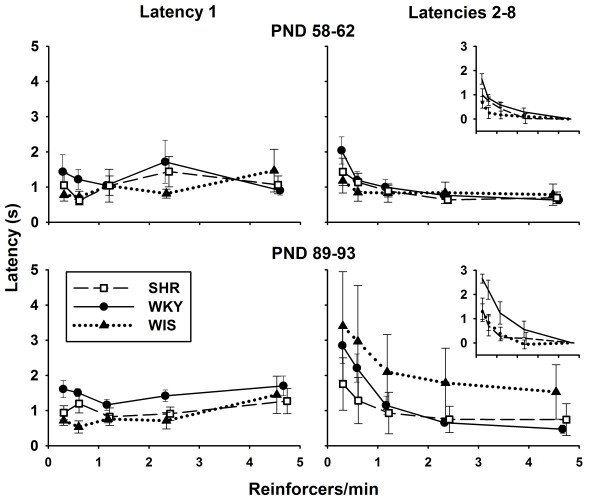
**Mean (± SEM) median Latency 1 (first latency within each VI schedule; left panels) and Latency 2-8 (right panels) as a function of mean rate of reinforcement, for each strain in epochs 1 and 2 (top and bottom panels, respectively)**. The insets of the right panels are mean rescaled latencies: each individual median Latency 2-8 in each schedule was divided by the median Latency 2-8 in VI 12 s of the same rat, and then logged (base 2). Thus, a rescaled latency of 3 indicates that the median latency in that VI (excluding Latency 1) was 2^3 ^= 8 times longer than in VI 12 s. For all strains, Latency 1 did not vary systematically with rate of reinforcement, whereas Latencies 2-8 were shorter with higher rates of reinforcement. SHR latencies were generally undistinguishable from those of the other strains, with the possible exception of the shorter SHR Latencies 2-8 when reinforcement was delivered less than once per minute.

Figure 4 shows mean (± SEM) median Latency 1 and Latencies 2-8 for each strain in each epoch, as a function of rate of reinforcement. Latency 1 (left panels) did not vary systematically with rate of reinforcement in either epoch or across strains in epoch 1. In epoch 2, mean median Latency 1 was longer for WKY than for the other strains, regardless of rate of reinforcement.

The right panels of Figure 4 and their insets show that Latencies 2-8 declined with rate of reinforcement. In epoch 1, Latencies 2-8 were mostly undistinguishable between strains, with the possible exception of the longer latencies of WKY at the lowest rate of reinforcement. In epoch 2, median Latencies 2-8 of WIS were longer on average, but also more variable across rats, than those of SHR and WKY. Also in this epoch, when rate of reinforcement was less than 1 per minute, mean median Latencies 2-8 were about 1 s shorter for SHR than WKY. The slopes of rescaled Latencies 2-8 (each median latency was divided by the median latency in VI 12 s, then logged, base 2), shown in the insets, reveal a within-subject sensitivity of Latencies 2-8 to rate of reinforcement in both epochs. This sensitivity was more pronounced in WKY than in the other strains.

### Inter-response times (IRTs): Bout-and-pause model

To account for the distribution of IRTs in each schedule, response rate in each VI schedule, excluding latencies, was disaggregated into bout-initiation rate (the reciprocal of the mean IRT separating response bouts) and within-bout response rate (the reciprocal of the mean IRT within bouts). This disaggregation consisted of estimating the parameters of a bi-exponential density function by fitting it to the distribution of IRTs in each VI schedule. The density function is

(3) $$p\left(IRT=t\right)=pwe^{-w\left(t-0.11\right)}+\left(1-p\right)be^{-b\left(t-0.11\right)},\;\;\;\;\;\;\;\;\;b\leq w\leq 9\;\text{s};0\leq p\leq 1$$

where *p *is the proportion of IRTs within bouts; 1/(1 - *p*) is the mean bout length, measured in lever presses. *w *is the response rate within bouts; *b *is the rate at which bouts are initiated. Because responses take a minimum time to be produced, that minimum time (the shortest possible IRT) must be subtracted from the duration *t *of every IRT [45]. The minimum IRT recorded for every rat was 0.11 s, which was the resolution at which responses were recorded. Therefore, 0.11 s were subtracted from *t *in the exponents of Equation 3, and *w *was constrained to be less than or equal to 1/0.11 ≈ 9 responses per second.

**Figure 5:**
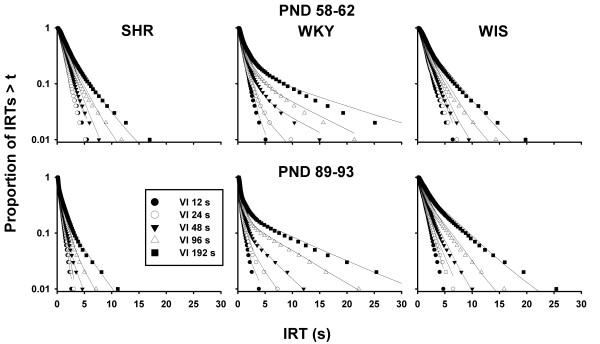
**Semi-log survival plots showing the mean proportion of IRTs greater than *t *in each schedule (symbols), strain (columns), and epoch (rows)**. Proportions were calculated for each rat in bins that contain, each, 1% of the IRTs; binned proportions were then averaged over rats. Curves through the data are the mean traces of the bout-and-pause model (Equation 3), drawn using maximally likely individual estimates. SHR survival functions (left panels) were steeper and more linear than those of WKY (center panels), indicating, respectively, shorter IRTs (higher response rate) and responses organized in less distinct bouts.

Parameters of Equation 3 were estimated for each individual rat in each epoch using the method of maximum likelihood [59]. Figure 5 shows semi-log survival plots of IRTs in each schedule and epoch, averaged within each strain. These plots have been used previously to illustrate differences in pause and bout responding in both rats and pigeons [42,60]. Often, these plots take on a "broken-stick" appearance with a steeply declining initial left limb and a more gradually declining right limb. A long initial limb on the leftmost side of the graphs indicates a high proportion of within-bout responses, *p*. The slope of the left limb is the within-bout rate of responding, *w*; the slope of the right limb is the rate of bout initiation, *b*. The curves in Figure 5 show that Equation 3 provided a good fit of the data, although the broken-stick pattern was most clearly visible in WKY at low rates of reinforcement.

**Figure 6:**
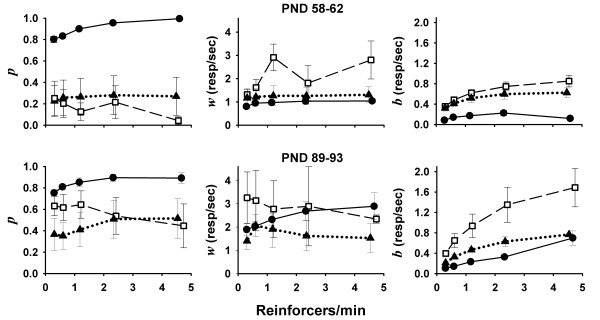
**Mean (± SEM) estimates of bout-and-pause parameters (Equation 3) as a function of mean rate of reinforcement, for each strain (SHR: squares; WKY: circles; WIS: triangles) in epochs 1 and 2 (top and bottom panels, respectively)**. In all epochs and schedules, SHR emitted shorter but more frequent bouts (low *p *in left panel, high *b *in right panel) than WKY. During epoch 1 SHR emitted faster within-bout responses (high *w *in top-center panel), but estimates of *w *were only possible for 2-3 out of 6 SHR rats; estimates of *w *were possible for all 6 WKY.

Mean (± SEM) bout-and-pause parameter estimates for each strain at each VI schedule and epoch are shown in Figure 6. To compute the mean estimates of *w *and *b*, individual estimates were weighed by *p *and (1 - *p*), respectively, because confidence on *w *and *b *estimates co-varies with these weights.^a ^The top panels of Figure 6 show the mean estimates of *p, w *and *b *in epoch 1; the bottom panels show estimates in epoch 2. Mean bout-and-pause parameter estimates are labeled in the same way as Herrnstein's hyperbola estimates (e.g., *p*_WKY_, *p*_SHR_, *p*_WIS_).

Estimates of the proportion of within-bout IRTs (*p*) in epoch 1 are shown in the top-left panel of Figure 6. Estimates of *p*_WKY _were substantially higher (mean across schedules = .90) than those of *p*_SHR _(.17) and *p*_WIS _(.26). This means that WKY produced substantially longer bouts than SHR and WIS. Moreover, whereas *p*_SHR _and *p*_WIS _were relatively constant across rates of reinforcement, *p*_WKY _increased with higher rates of reinforcement, from .80 at the lowest rate to .99 at the highest rate.

Estimates of within-bout response rate (*w*) in epoch 1 are shown in the top-middle panel of Figure 6. Estimates of *w*_WKY _and *w*_WIS _increased only slightly with rate of reinforcement. At the lowest rate of reinforcement, *w*_WKY _= 0.80 and *w*_WIS _= 1.17 responses per second; at the highest rate of reinforcement, *w*_WKY _= 1.04 and *w*_WIS _= 1.31 responses per second. These trends were dwarfed by the large between-subject and between-schedule variability in estimates of *w*_SHR_. Moreover, in every schedule, *w*_SHR _>*w*_WIS _>*w*_WKY _(mean across schedules = 2.09, 1.24, and 0.96 responses per second, respectively). It is important to note, however, that estimates of *w*_SHR _and *w*_WIS _were based on 2-3 rats of each strain, because *p *= 0 for most of these rats in most schedules.

Estimates of bout initiation response rate (*b*) in the first epoch are shown in the top-right panel of Figure 6. Estimates of *b*_SHR _and *b*_WIS _systematically increased with rate of reinforcement. At the lowest rate of reinforcement, *b*_SHR _= 0.36 and *b*_WIS_= 0.32 responses per second; at the highest rate of reinforcement, *b*_SHR _= 0.85 and *b*_WIS_= 0.63 responses per second. Estimates of *b*_WKY_varied as an inverted-U function of rate of reinforcement, peaking at the second highest rate of reinforcement (0.23 responses per second). In every schedule, *b*_SHR _>*b*_WIS_>*b*_WKY_(mean across schedules = 0.61, 0.49, and 0.15 responses per second, respectively).

The bottom panels of the Figure 6 show the mean (± SEM) estimates of *p, w *and *b *in epoch 2. The bottom-left panel of Figure 6 shows that, similar to those in the preceding epoch, estimates of *p*_WKY _in epoch 2 were very high and increased further with rate of reinforcement. Estimates of *p*_SHR _and *p*_WIS _increased between epochs in every VI schedule. The increase was particularly noticeable in *p*_SHR_; on the average, estimates of *p*_SHR _more than tripled between epochs. Like in the preceding epoch, however, there were no trends in *p*_SHR _and *p*_WIS _across VI schedules comparable to those of *p*_WKY_.

Estimates of *w *in epoch 2 are shown in the bottom-middle panel of Figure 6. Estimates of *w*_WKY _and *w*_WIS _increased between epochs in every schedule, but only *w*_WKY _preserved its positive correlation with rate of reinforcement. Estimates of *w*_SHR _remained relatively high, particularly when rate of reinforcement was low. Between-subject variance in individual estimates of *w*_SHR _increased substantially between epochs, further dwarfing any differences between strains. The increase in between-subject variance was due to 2 SHR with undetermined *w *in epoch 1, whose individual *w *estimates, averaged over VI schedules in epoch 2, were 5.50 and 8.89 responses per second. Such high estimates were not obtained for any other rat of any strain.

Estimates of *b *in the second epoch are shown in the bottom-right panel of Figure 6. As in the preceding epoch, *b*_SHR _>*b*_WIS _≥ *b*_WKY _in every VI schedule, and estimates of *b *also increased with rates of reinforcement, including *b*_WKY_. Estimates of *b*_SHR _and *b*_WKY _increased between epochs in every VI schedule; *b*_WIS _remained relatively unchanged.

## Discussion

Operant hyperactivity was observed in SHR, particularly during adulthood (epoch 2, PND 89-93), but only at low rates of reinforcement (less than 2 and 4 reinforcers per minute on PND 58-62 and 89-93, respectively). Estimates of Herrnstein's hyperbola parameters (*k, R_e_*; see Equation 1) suggest that operant hyperactivity in SHR is not due to enhanced motor capacity relative to WKY (the converse is most likely the case: *k*_WKY _>*k*_SHR_). Instead, highly valued activities-such as searching for food-are less likely to be displaced by less valued, competing activities in SHR than in WKY (*R*_*e*SHR_<*R*_*e*WKY_). This finding is consistent with the notion that frequent reinforcement normalizes free-operant ADHD performance [36,37].

Differences between WKY and SHR in response rate and in estimates of Herrnstein's hyperbola parameters replicate prior findings in adult rats [32,33], and generalize them, to a limited extent, to younger rats. Such generalization suggests that the inferences drawn from the more detailed analysis, based on bout-and-pause parameter estimates, are not idiosyncratic to the present data, but reflect a more general phenomenon. Unlike Herrnstein's hyperbola, the bout-and-pause model does not assume the functional equivalence of all operant responses. Instead, the bout-and-pause model supports an analysis based on latency and IRT statistics that separates responses into more meaningful functional categories, which we examine next.

### Latencies

Latencies shown in Figure 4 suggest that tones did not support the discrimination between schedules of reinforcement. Instead, it appears that latencies were updated according to preceding intervals to reinforcement. Such a process is evinced in the steeper Latency 2-8 curves (right panels) relative to Latency 1 curves (left panel). When the interval to reinforcement was not cued by prior intervals (Latency 1), WKY took longer to emit the first response, relative to SHR and WIS, but only in adulthood. In subsequent intervals (Latencies 2-8), WKY latencies became particularly sensitive to rate of reinforcement, especially in adulthood. Note that, in adulthood, the pattern of Latencies 2-8 (bottom-right panel) is a vertically-flipped analogue of response rates (Figure 2, bottom panel). This indicates that adult Latencies 2-8 became either (1) an important determinant of response rate, or (2) sensitive to whatever factors determined response rate. Alternative (1) does not appear to be the case: based on mean adult performance of each strain, for every latency there were about 11 - 20 IRTs in VI 12 s, and up to 58 - 150 IRTs in VI 192 s. That is, latencies contributed between 0.67% and 8% of the response rate denominator. Although latencies appear to reflect patterns of hyperactivity in SHR, particularly in adulthood, they cannot be the main source of these patterns. That source is, therefore, most likely to be identified in the distribution of IRTs.

### IRTs

The bout-and-pause model assumes that responses are organized in two separate categories: responses that initiate bouts and responses emitted within bouts. An analysis based on bout-and-pause premises suggests that SHR hyperactivity reflects a high rate of bout initiations (higher *b*) in SHR relative to WKY (Figure 6, right panels), even though SHR response bouts were shorter (lower *p*; Figure 6, left panels). Because of the short length of their bouts, estimates of SHR within-bout response rate (*w*) were not reliable. Nonetheless, the performance of those SHR with *p *> 0 suggests that SHR within-bout response rates were higher than those of the other 2 strains, particularly in epoch 1. SHR bouts became longer in adulthood, but were still systematically shorter than those of WKY, regardless of schedule. The length and density of WKY bouts, unlike those of other strains, increased with increasing rate of reinforcement, which may explain why differences in overall response rate between SHR and WKY are confined to low rates of reinforcement. In epoch 1, WIS parameters were generally intermediate to those of SHR and WKY across parameters; in epoch 2, WIS maintained relatively short bouts that contained few responses.

The bout-and-pause analysis thus identifies the higher frequency of bouts in SHR as the main source of hyperactivity, and higher within-bout rate as a possible secondary source. Rate of bout initiation is particularly sensitive to motivational manipulations, increasing as a function of reinforcement deprivation and availability in rats [42-45], mice [61], and pigeons [62]. This correlation suggests that SHR hyperactivity is caused by a hypermotivation to incentives. Such inference is consistent with latency patterns in adulthood, to the extent that latencies are indicative of motivation [63,64], and with inferences drawn from Herrnstein's hyperbola parameters, both here and in prior studies [32,33].

### A delay-of-reinforcement-gradient hypothesis

Brackney and colleagues [45], however, caution against a straightforward interpretation of changes in rate of bout initiation in terms of incentive motivation. Based on the performance of Sprague-Dawley rats, they concluded that changes in bout initiation rate *alone *may be interpreted as changes in incentive motivation, but when such changes are accompanied by changes in other parameters, they may reflect non-motivational processes that only indirectly impact motivation. For instance, a tandem ratio requirement at the end of the VI lengthens bouts and increases the number of responses within them, but also reduces the frequency of bouts [42-45]. The latter effect cannot be accounted for by a reduction in incentive motivation, because the tandem requirement does not change the rate of reinforcement substantially. Instead, Brackney and colleagues suggested that the tandem requirement favors the reinforcement of long response bouts [65], concomitantly reducing the temporal contiguity between bout initiations and reinforcement. It is hypothesized that reduced initiation-reinforcement contiguity results in less effective reinforcement of bout initiation and a consequent reduction in its rate.

**Figure 7:**
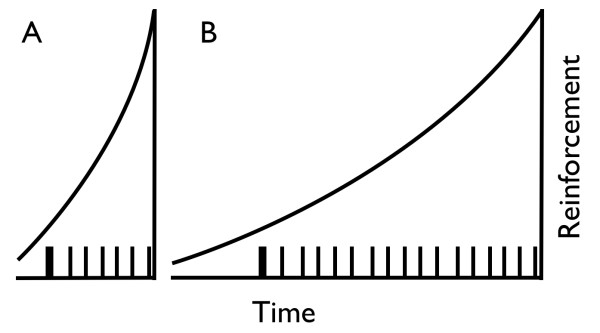
**Delay-of-reinforcement-gradient hypothesis of SHR hyperactivity**. Ticks on the *x*-axis are responses; thick ticks are bout initiations. A reinforcer is delivered after the last response on the right of each panel. The sloped curves indicate that reinforcement is more effective with temporal proximity to the reinforcer. For SHR, only short bouts are effectively reinforced (panel A); for WKY, longer bouts are reinforced (panel B). Note that reinforcement affects more behaviors in panel B than in panel A, which entails that a smaller proportion of reinforcement strengthens bout initiation in panel B than in panel A. Therefore, relative to competing behaviors such as activities between bouts and responses within bouts, bout initiations are less effectively reinforced when reinforcement gradients are flatter.

Brackney and colleagues' [45] account of how a tandem requirement reduces bout initiation rate implies that the effectiveness of reinforcement declines with the temporal distance between the reinforced response (bout initiation) and the reinforcing event (food) [66,67]. The slope of this decline in reinforcement effectiveness is the *delay-of-reinforcement gradient *[68,69]. The effect of flattening the delay-of-reinforcement gradient on operant performance should be similar to the effect of imposing a tandem requirement: It should facilitate the reinforcement of long bouts while reducing bout frequency. Compared to the initiation of short bouts supported by steeper gradients, the initiation of long bouts supported by flatter gradients should be less frequent because flatter gradients envelop more competing responses (between-bout activities, within-bout responses) than steeper gradients. These intuitions are diagrammed in Figure 7.

Compared to SHR, WKY displayed long-but-infrequent response bouts (high *p *and low *b *in Figure 6) in both assessment epochs. This pattern suggests that one important source of SHR hyperactivity is the steepness of its delay-of-reinforcement gradient. This hypothesis is consistent with prior SHR data [34,38,70-73] and with observations of children with ADHD [74]. The characterization of ADHD in terms of steeper delay-of-reinforcement gradients is a core assumption of the dynamic developmental theory of ADHD [38,75,76]. Regarding WIS rats, the intermediate length (in epoch 1) and frequency (in both epochs) of their bouts suggest an intermediate delay-of-reinforcement gradient for this strain relative to SHR and WKY.

The emission of short free-operant bouts, which supports the delay-of-reinforcement-gradient hypothesis of operant hyperactivity, has recently been observed in children with ADHD [77]. The critical evidence was collected using concurrent independent VI schedules of reinforcement, where two schedules, like those used in the present study, were simultaneously in effect In this context, Shull [47] has argued that the length of a "visit" to either schedule is functionally equivalent to the length of bouts in a single-schedule design (following Herrnstein's rationale, single-schedule designs may be thought of as concurrent-schedule designs where one schedule is implicit [40]). Taylor, Lincoln and Foster [77] reported that children with ADHD switch more between concurrent VI schedules, and thus produce shorter visits, than non-ADHD controls, as long as switching between schedules is not penalized with a changeover delay. The converging patterns of free-operant performance in SHR and in children with ADHD suggest that (1) the emission of short free-operant bouts may be a diagnostic feature of the behavioral phenotype of ADHD, revealing a deeper deficit in learning response-reinforcement contingencies, and that (2) the SHR models these attributes of ADHD, further confirming its utility as an animal model of ADHD [13].

### Alternative sources of hyperactivity

SHR produced shorter bouts at a higher rate than WKY over the range of VI schedules tested in the present study. Prior research [42-45] suggests an interpretation of these differences in terms of delay-of-reinforcement gradients. Based on such interpretation, it would be expected that appending a tandem ratio requirement to the VI schedule of SHR would reduce the difference in its performance relative to WKY. But aside from lengthening bouts and reducing their frequency, a tandem requirement also increases within-bout responding, which has the net effect of increasing overall response rate [42,44,45]. That is, the tandem-ratio "treatment" is expected to increase SHR activity, not decrease it. This means that, although steeper delay-of-reinforcement gradients may be the main source of SHR overactivity, it is unlikely to be the only one. Two additional sources are possible:

#### Increased motor capacity in SHR

Estimates of Herrnstein's hyperbola parameters ruled out motor capacity as a source of SHR hyperactivity, on the basis of projected asymptotic response rates (*k*). It appears intuitive that such asymptotic rates reflect motoric constraints in performance. Nonetheless, SHR and WIS within-bout responses (the faster response class) constituted only about half of the responses at the highest rate of reinforcement, when response rates were nearly asymptotic. This means that SHR and WIS could respond faster than what Herrnstein's *k *suggests. Parameter *w *is probably a more realistic reflection of motoric constraint.^b ^Estimates of the highest within-bout response rate across schedules (Figure 6, middle panels) suggest somewhat higher motoric capacity in young SHR (PND 58-62), and limited motoric capacity in adult WIS (PND 89-93). Although estimates of *w *are compromised by the short bouts produced by SHR and WIS, elevated motoric capacity may not be ruled out as a potential source of SHR hyperactivity, at least not during the transition from adolescence to adulthood. Furthermore, the notion that motoric capacity is involved in SHR hyperactivity is consistent with prior data showing that IRTs shorter than 0.4 s are more frequently emitted by adult SHR than by adult WKY in VI 30 s [34].

#### SHR hypermotivation

Although the interpretation of differences in bout-initiation rate (*b*) in terms of incentive motivation is conditional to the absence of changes in other parameters (see *A delay-of-reinforcement-gradient hypothesis*), changes in *p *and *w *do not rule out differences in incentive motivation. In fact, it appears that the reduction in *b *that is expected from a tandem ratio treatment would be too small to reduce SHR estimates to WKY levels: Brackney and colleagues [45] report that a tandem ratio requirement reduced *b *by 37% in a VI 120 s. In a comparable schedule (VI 96 s), estimates of *b *in WKY were 71% shorter than SHR in epoch 1, and 78% shorter in epoch 2. Therefore, it seems likely that incentive motivation differences in *b *by itself-also contributed to operant hyperactivity in SHR.

### Limitations

Finally, we acknowledge and address three potential limitations of the present study. These limitations do not compromise the basic conclusions inferred from the data, but constrain the interpretation of the present results in terms of underlying psychological and developmental processes.

#### Lack of stimulus control

The first latency in each VI component (Latency 1 in Figure 4) did not vary systematically with rate of reinforcement for any strain. This indicates that the tone associated with each VI schedule was not effective in controlling response rate. Differences in response rate across schedules depended on adjustments of response rate to local rate of reinforcement. Such adjustments may have introduced extraneous variability in VI performance among strains. Furthermore, even if the tones had been effective discriminative stimuli, schedule interactions might have also confounded our results. These limitations, however, do not appear to seriously compromise the findings of the present study, for two reasons. First, the changes in overall response rate as a function of rate of reinforcement and strain resemble those observed before [32,33], which were not collected in a multiple-schedule context. Such close resemblance suggests that differences between SHR and WKY performance are robust against confounding factors in the present study. Second, the critical differences in bout-and-pause parameters between strains (high *p *in WKY, high *b *is SHR) were not schedule-dependent.

#### Differentiation between within-bout response rate and bout-initiation rate

Of the 90 rat × schedule estimations of bout-and-pause parameters in each epoch, 46 in epoch 1 and 20 in epoch 2 yielded *p *= 0 or 1. In those cases, the distribution of IRTs did not resemble a mixture of two exponentials (Equation 3) but just a single exponential. This is noticeable in the nearly linear (in logarithmic scale) IRT survivor plots shown in Figure 5, particularly those of SHR and WIS and of rich schedules. Exponential IRT distributions yielded ambiguous estimates of *p *and indeterminate estimates of either *w *or *b *(footnote 1 clarifies how it was chosen between *p *= 0 and *p *= 1 in each estimation). Despite consistent differences in parameters across schedules and strains, the uncertainty regarding parameter estimates implies that inferences drawn from them, particularly in epoch 1, should be taken with caution. Although the short length of SHR bouts is itself a very important finding, future research should promote longer bouts by imposing small tandem ratio requirements to all strains. This methodological adjustment would make pauses between and within bouts more readily distinguishable.

#### Confound of training experience and age

The parameters of Herrnstein's hyperbola and the bout-and-pause model were examined in two epochs, PND 58-62 and 89-93. Between epochs, response rates increased across schedules in SHR, only at high reinforcement rates in WKY, and not visibly in any schedule in WIS. These divergent patterns of change over time exacerbated the differences in response rate between SHR and control strains at low rates of reinforcement. The elevated rate of weakly reinforced responses is the signature of operant hyperactivity in SHR [32,33]. One possible implication of the present results is that hyperactivity emerges more strongly with adulthood. Although past research is consistent with these results [34], they do not appear to be consistent with the modal developmental trajectory of hyperactivity in ADHD [78-80]. Impulsive-hyperactive symptoms associated with ADHD generally decline with age. Note, however, that the present study did not examine age separately from training experience: older, more hyperactive SHR had more exposure to the schedules of reinforcement than younger, less hyperactive SHR. The inconsistency between hyperactivity in SHR and in ADHD may stem from this confound. Our data, in fact, points at a possible coincidence between the developmental trajectories of SHR and ADHD hyperactivity: SHR learned to (or matured to, we do not know) produce longer response bouts, a pattern that was more typical of WKY controls. Although this study was not primarily aimed at discriminating between practice and maturational effects on operant hyperactivity, it provides, nonetheless, hints that may guide future research on developmental factors involved in ADHD.

## Conclusions

This study confirms that operant hyperactivity in SHR, a purported animal model of ADHD, is expressed only at low rates of reinforcement. This effect was observed in the transition from adolescence to adulthood (PND 58-62) and, more markedly, during early adulthood (PND 89-93). A close examination of the microstructure of VI performance indicates that, across ages and schedules, operant hyperactivity in SHR may be due to steeper delay-of-reinforcement gradients relative to control strain WKY. Inordinate motivation for incentives and elevated motoric capacity may also contribute to operant hyperactivity in SHR. With adulthood, delay-of-reinforcement gradients in SHR appear to flatten; its motoric capacity becomes hardly distinguishable from WKY, but its motivation for highly valued incentives, such as sucrose pellets, grows even stronger. Whether these changes in performance parameters are due to training experience, maturation, or a combination of both, is yet unclear. These results suggest, nonetheless, that complex and important learning, motivational, and developmental processes expressed in SHR behavior appear to underlie operant hyperactivity in ADHD.

## Appendix: Computing mean response rate from its components

Response rate *B *over an interval *T *is the number of responses made in that interval (*N*) divided by *T*. Therefore,

(A1) N=BT

If *T *is the interval between trial onset and reinforcement, then *T *may be partitioned into two periods: the time between trial onset and the first response (latency, or *L*) and the time between the first response and the reinforced response (*T *- *L*). The latter may be further portioned out into *N *- 1 inter-response times (IRTs). The mean IRT is *t *= (*T *- *L*)/(*N *- 1). Solving for *T *in the mean IRT equation and then substituting *N *with *BT*,

(A2) T=L+(BT-1)t;

solving for *B*,

(A3) $$B=\frac{1}{T}+\frac{1}{t}-\frac{L}{Tt}.$$

Based on the assumed distribution of IRTs (Equation 3), and assuming a minimum response duration δ (0.11 s in this report), the mean IRT is

(A4) $$t=\frac{p}{w}+\frac{\left(1-p\right)}{b}+\delta .$$

Mean response rate in a VI schedule may thus be recovered by substituting *t *in Equation A3 with the right-hand side of Equation A4, and assuming that *T *equals the VI requirement *I*. A more precise estimate of *T *is

(A5) $$T=I+\frac{t}{2}.$$

## Endnotes

^a^The estimate of *p *for several rats under various VI schedules was 1.0 or zero, which posed a problem for parameter estimation. Whether *p *= 1.0 or zero, or *w *= *b*, Equation 1 is reduced to an exponential density function, with either *p *= 1.0, and *b *not computable (i.e., bouts are infinitely long) or *p *= 0, and *w *not computable (i.e., bouts are 1 lever press long). These two situations are not distinguishable. When *p *= 1.0 or *p *= 0 had to be chosen for a particular rat, the variance with respect to *p *estimates in other VI schedules within the same subject were taken into consideration. The estimate of the ambiguous *p *was the one that minimized the variance among *p *estimates. When *p *could take either value, 1.0 or zero, in all 5 VI schedules (this happened in 7 of 36 rat × epoch observations), *p *was invariably estimated to be zero, because under such assumption the mean estimate of *b *for these rats (0.54 resp/sec) was closer to the mean estimate of *b *for other rats and epochs (0.42 resp/sec) than to the estimates of *w *for other rats and epochs (2.28 resp/sec). ^b^Brackney and colleagues (2011) considered δ, the shortest possible IRT, a better estimation of motoric constraint than *w*. Because of the low temporal resolution at which responses were recorded in the present study (9 Hz), δ could not be analyzed separately. It is likely, however, that variations in *w *between strains comprise variations in δ, particularly at high values of *w*.

## List of Abbreviations

ADHD: Attention deficit hyperactivity disorder; SHR: Spontaneously hypertensive rat; WKY: Wistar Kyoto rat; WIS: Wistar rat; VI: Variable interval; IRT: Inter-response time; AICc: Corrected Akaike Information Criterion.

## Competing interests

The authors declare that they have no competing interests.

## Authors' contributions

FS conceived and designed the study. JH and KH acquired and analyzed data. JH, KH and FS interpreted data and drafted and revised the manuscript. Parts of the manuscript served as Honors thesis for KH. All authors read and approved the final manuscript.
